# Evolving Role of Lymphedema Surgery on Breast Reconstruction: A Systematic Review and Multi-Institutional Algorithmic Approach

**DOI:** 10.3390/jcm13216518

**Published:** 2024-10-30

**Authors:** Min-Jeong Cho, Jorge Flores Garcia, Yujin Myung, Han Gyu Cha, Akitatsu Hayashi, Joon Pio Hong, Roman Skoracki

**Affiliations:** 1 Department of Plastic and Reconstructive Surgery, The Ohio State University Wexner Medical Center, Columbus, OH 43201, USA; 2 Department of Plastic and Reconstructive Surgery, Seoul National University Bundang Hospital, Seoul National University College of Medicine, Seongnam 03080, Republic of Korea; 3 Department of Plastic and Reconstructive Surgery, Soonchunhyang University Bucheon Hospital, Bucehon 14584, Republic of Korea; 4 Lymphedema Center, Department of Breast Center, Kameda Medical Center, Chiba 296-0041, Japan; 5 Department of Plastic and Reconstructive Surgery, Asan Medical Center, Seoul 05505, Republic of Korea

**Keywords:** lymphedema, BCRL, breast reconstruction, VLNT, LVB, lymphatic surgery, breast cancer

## Abstract

**Background/Objectives**: Recent advancements in breast cancer treatment have led to increased survival rates, prompting a shift towards addressing breast cancer-related lymphedema (BCRL). Despite the evolving role of lymphatic surgery in breast reconstruction, there is limited literature evaluating the current role of lymphatic surgery in breast reconstruction. This review aims to evaluate the state of lymphatic surgery in breast reconstruction, analyzing surgical techniques and proposing a multi-institutional algorithmic approach. **Methods**: Through a search and screening of literature, data regarding the study type, type of operation (bypass, pLVB/ILR/LYMPHA, VLNT, or a combination of treatments), and clinical outcomes were collected. **Results**: The systematic review included 184 studies. Overall, the number of publications on lymphatic surgery increased from 4.4 per year (2010–2016) to 21.1 per year since 2017. The most published procedure was vascularized lymph node transfer (34.6%), followed by preventive lymphatic surgery (31.4%), therapeutic lymphovenous bypass (23.3%), and combined breast and lymphatic reconstruction (10.7%). While VLNT was the most published procedure, preventive surgery has been the most published topic since 2020, with 11.7 articles per year since. Similarly, there has been an increase in studies on combined lymphatic surgery and breast reconstruction in the last five years, with 16 articles published. **Conclusions**: The role of lymphatic surgery in breast cancer patients is evolving, with an increasing emphasis on preventive procedures and combined reconstructive approaches. However, our study shows that the current literature is predominantly based on lower-level evidence, highlighting the need for more randomized controlled trials to establish stronger clinical recommendations.

## 1. Introduction

Recently, there have been various paradigm shifts in the field of breast reconstruction due to technological and pharmacological advances leading to increased breast cancer patient survival. Plastic surgeons are now faced with patients who will undergo or have undergone locally aggressive and systemic therapy for the treatment of axillary lymph node metastasis, which significantly increases their risk for the development of breast cancer-related lymphedema (BCRL). BCRL is a chronic, debilitating condition characterized by chronic swelling of the affected extremity due to the disruption of the lymphatic system, commonly by axillary lymph node dissection, radiation, and/or taxol therapy. It is estimated that 1 in 5 breast cancer patients suffer from BCRL, which leads to significant negative psychological, emotional, and financial impacts on survivors [1].

Despite the evolving role of lymphatic surgery on breast reconstruction, there is limited literature evaluating the current role of lymphatic surgery in breast reconstruction. Plastic surgeons now offer a myriad of reconstructive options, including prophylactic lymphovenous bypass (pLVB), lymphovenous anastomosis/bypass (LVA/LVB), vascularized lymph node transfer (VLNT), and combined lymph node transfer and autologous-based breast reconstruction [2,3,4,5,6]. While there has been a significant increase in the amount of literature due to the importance of lymphatic surgery, it is important to understand the current state of lymphatic procedures at various stages of breast reconstruction. Therefore, we performed a systematic review of the literature on this topic, and we present our multi-institutional approach toward expanding the role of lymphatic surgery in breast reconstruction.

### 1.1. Current State of Lymphatic Surgery in Breast Cancer Patients

#### 1.1.1. Physiologic Surgery: Lymphovenous Anastomosis/Bypass

Currently, lymphovenous anastomosis (LVA), also referred to as lymphovenous bypass (LVB), is the first line of treatment in patients with early-stage lymphedema. In this procedure, a supermicrosurgical anastomosis is performed between a functional lymphatic vessel identified by Indocyanine green (ICG) lymphangiography and a recipient’s vein to re-establish lymphatic drainage in the affected areas. It offers several advantages, including minimal invasiveness, short-term recovery, and high success rates [7]. While many studies have demonstrated the efficacy of the procedure, it has historically been limited to patients with an early stage of lymphedema as the depth penetration of the ICG lymphangiography is limited to 1–2 cm, limiting its utility in patients with skin fibrosis [8].

#### 1.1.2. Physiologic Surgery: Vascularized Lymph Node Transfer

Historically, the vascularized lymph node transplantation (VLNT) procedure is reserved for patients who show minimal improvement after lymphovenous anastomosis or in those with advanced stages of lymphedema [9,10]. In this procedure, lymph node tissue from a different part of the body is transplanted to the lymphedematous area along with blood vessels, akin to a form of free flap surgery [11]. The proposed mechanism of action of VLNT is via lymphatic pumping and lymphangiogenesis mediated by growth factors [12,13,14]. Currently, lymph nodes for VLNT can be harvested from submental, supraclavicular, inguinal, lateral thoracic, and omental regions, chosen based on factors such as the site of lymphedema, patient preference based on scar location, and surgeon preference. For patients with BCRL, the majority of microsurgeons utilize three donor sites for VLNT: the omental flap, the deep inferior epigastric perforators (DIEP) flap with either the superficial inferior epigastric artery (SIEA) or the superficial circumflex iliac artery (SCIA)-based lymph node flap, and the superficial circumflex iliac artery perforator (SCIP) flap.

#### 1.1.3. Preventive Lymphatic Surgery

In 2009, Boccardo et al. introduced the lymphedema microsurgical preventive healing approach (LYMPHA), where ligated lymphatic vessels are intussuscepted into the tributary of the axillary vein at the time of axillary lymph node dissection (ALND) to prevent the development of BCRL [15]. Since then, several reports on BCRL prevention have described the effect of immediate lymphatic reconstruction (ILR) and prophylactic LVB (pLVB), which utilize surgical techniques similar to the LYMPHA [16,17,18,19,20]. However, as the cumulative incidence of BCRL is known to increase over time until 24 months after surgery [21,22], long-term cumulative results of these prevention techniques are essential to evaluate their effects. A recent randomized controlled trial demonstrated the preliminary results of a 9.5% cumulative incidence of BCRL in the immediate lymphatic reconstruction group compared with 32% in the control group at a 2-year follow-up [23]. Currently, there are two other reports demonstrating contrary, long-term incidence data at the 4-year follow-up, where one presented a BCRL incidence of 31.1% in patients who underwent LYMPHA [24], while the other resulted in 9% following ILR at the 4-year study period [25]. This discrepancy in long-term benefit may be a result of the specific preventive procedure performed; however, to our knowledge, there is no present study comparing the efficacy of the techniques. Lastly, some review studies with meta-analyses have also shown promising results in reducing the rate of secondary lymphedema in both upper and lower extremities [26,27].

#### 1.1.4. Concurrent Breast Reconstruction and Lymphedema Surgery

While early studies have indicated the benefit of immediate breast reconstruction in reducing the occurrence of BCRL, it has been debated whether autologous tissue-based breast reconstruction without concurrent lymph node transfer truly leads to improvement in lymphedema patients. In addition, there is no consensus on the timing and ideal combination of VLNT and breast reconstruction [28,29,30]. Therefore, many plastic surgeons now employ a patient-tailored approach, including staged procedures, different combinations of autologous free flap and VLNT, or implant-based reconstruction with lymphatic procedures. Due to the variations in the type of treatment, there are no high-level studies comparing their outcomes.

### 1.2. Institutional Algorithm

#### 1.2.1. Breast Cancer-Related Lymphedema Prevention

**Figure 1:**
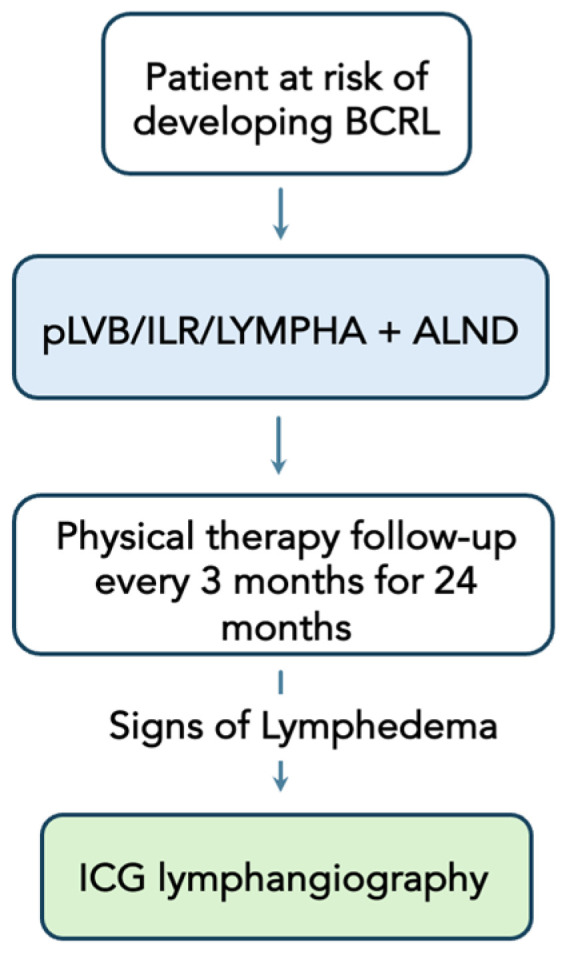
Breast cancer-related lymphedema prevention program. BCRL = breast cancer-related lymphedema; LYMPHA/ILR/pLVB = lymphatic microsurgical preventive healing approach/immediate lymphatic reconstruction/prophylactic lymphovenous bypass; ALND = axillary lymph node dissection; ICG = Indocyanine green.

All of the authors’ institutions have a multi-disciplinary lymphedema program, which collaborates closely with certified lymphedema therapists, surgical oncologists, and nutritionists. Patients who are at risk of developing BCRL due to ALND will be placed in a lymphedema surveillance program, which involves lymphedema preventive surgery (ILR, LYMPHA, and pLVB) at the time of ALND and a close follow-up with physical therapy every 3 months for 24 months after surgery. Any patients with the symptoms of lymphedema (heaviness, swelling, cellulitis, etc.) and/or significant changes in the measurements concerning lymphedema development will undergo ICG lymphangiography for investigation (Figure 1). Then, patients will undergo the following algorithm based on the severity of their lymphedema and desire for breast reconstruction.

#### 1.2.2. Breast Reconstruction in Patients with Breast Cancer-Related Lymphedema

**Figure 2:**
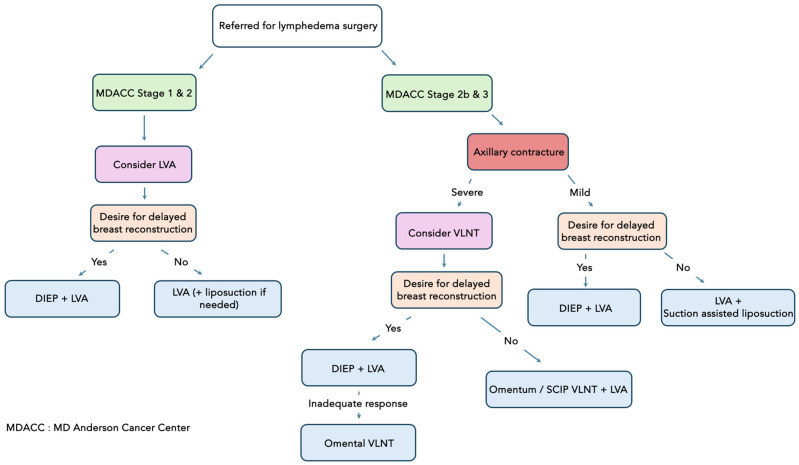
Multi-institutional lymphatic and breast reconstruction in patients with BCRL. VLNT = vascularized lymph node transfer; LVA= lymphovenous anastomosis; DIEP = deep inferior epigastric artery perforator.

The goal of breast reconstruction and lymphedema surgery in BCRL patients includes not only the relief of lymphedema but also restoring functional and aesthetically pleasing breasts if desired. In our institutions, we employ a patient-tailored approach toward BCRL patients while carefully considering patients’ current lymphatic status and aesthetic goals based on institutional experiences (Figure 2). First, patients with BCRL will be stratified based on their MD Anderson Cancer Center (MDACC) ICG lymphography stage. For the patients with early-stage BCRL (MDACC stages 1 and 2) who do not desire any breast reconstruction, they will undergo LVB. If patients have advanced-stage lymphedema (MDACC stages 2b and 3), then patients can benefit from either the placement of an omental flap in the axilla by anastomosing the flap to the thoracodorsal artery or one of its branches post-axillary scar release. Unlike supraclavicular, submental, or groin flaps, which can lead to iatrogenic lymphedema or chyle leak, iatrogenic lymphedema following omental flap harvest has not been reported. However, disadvantages of this procedure include the need for intra-abdominal surgery, associated complications, and potentially noticeable abdominal scars. Additionally, harvesting lymph nodes in a relatively unfamiliar area may present a challenge for plastic surgeons and require co-surgeoning with general surgeons. When intact and functional lymphatic vessels are identified via pre-operative imaging, LVA is performed in conjunction with VLNT.

If patients do desire breast reconstruction, the timing of the surgery is based on the patient’s preference regarding the donor site of the VLNT and the severity of their lymphedema. First, autologous-based breast reconstruction, such as the deep inferior epigastric artery perforator (DIEP) flap, profunda artery perforator (PAP) flap, lumbar artery perforator (LAP) flap, etc., would be performed in conjunction with LVB in patients with early-stage lymphedema. For patients with late-stage lymphedema, then they would undergo autologous-based reconstruction + LVA, followed by omental VLNT as needed.

## 2. Materials and Methods

### 2.1. Search Strategy

To investigate the role of lymphatic surgery in breast reconstruction, the Preferred Reporting Items for Systematic Reviews and Meta-Analyses (PRISMA) guidelines for systematic reviews were followed to report our findings. We performed a systematic review of the Academic Search Premier and MEDLINE databases via EBSCOHost in January 2024 using the following search terms: “lymphedema AND breast”, “lymphedema AND upper extremity”, combined with “lymphovenous bypass”, “lymphovenous anastomosis”, “lymphaticovenous”, “lymph node transfer”, “immediate lymphatic reconstruction”, and “LYMPHA”.

### 2.2. Study Selection, Data Extraction, and Level of Evidence Analysis

**Figure 3:**
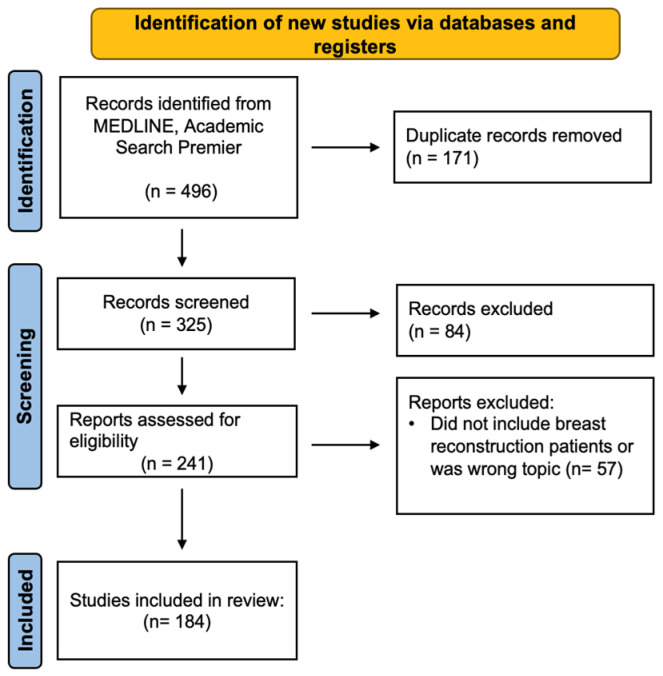
Preferred reporting items for systematic reviews and meta-analyses (PRISMA) flow diagram.

To identify comprehensive trends in the literature, we included articles from all levels of evidence published prior to 2024, except commentary and editorial pieces. The exclusion criteria included non-English and non-human articles as well as studies exclusively on lower-limb lymphedema, non-surgical management of BCRL, and those not concerned with the surgical component of lymphatic surgery (i.e., economic factors). Screening of titles and abstracts, text review, and extraction of data were performed by one author (JFG). The included articles were individually analyzed and sorted based on the study type and type of operation discussed (bypass, pLVB/ILR/LYMPHA, and VLNT, or a combination of treatments). Review articles on surgical management of lymphedema that were not exclusive to one procedure were placed in a separate category. Data on the surgical methodology and clinical outcomes were extracted from the studies providing such (Figure 3). Then, a level of evidence analysis was assigned to each article using the American Society of Plastic Surgeons Level of Evidence rating scale [31] (Table 1).

## 3. Results

### 3.1. Systematic Literature Review

**Figure 4:**
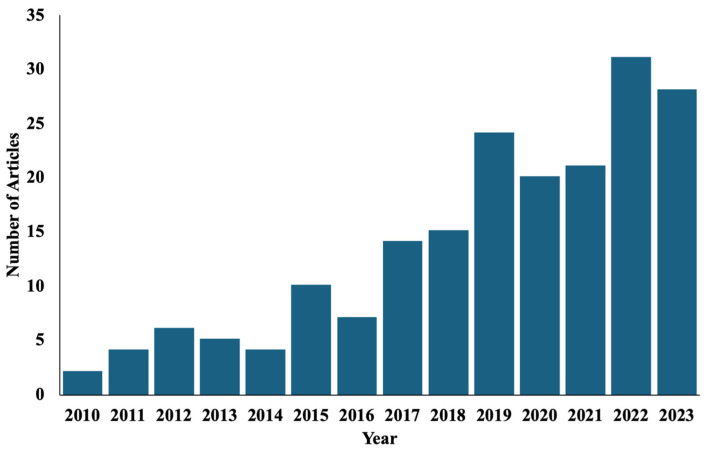
Trend of studies on breast cancer-related lymphedema surgical management from 2010 to 2023.

**Figure 5:**
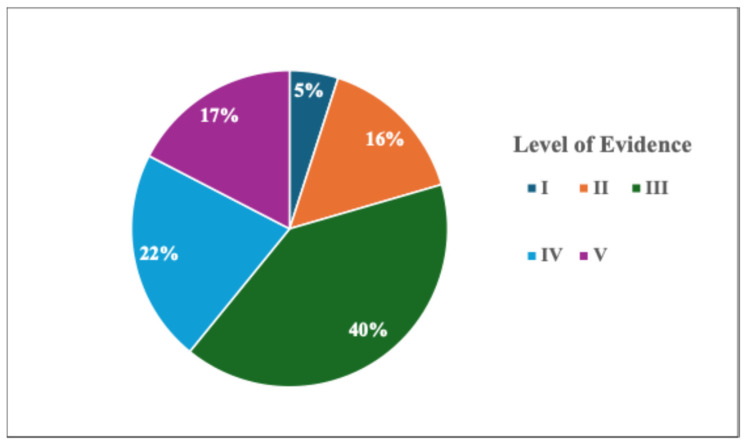
Analysis of studies based on level of evidence.

Our initial search yielded 184 studies of potential relevance (Table 2) and revealed that there has been a significant shift in the number of articles being written on lymphatic surgery in breast cancer patients from 4.4 per year from 2010 to 2016 to 21.1 per year since (Figure 4). Of these, the most published lymphatic procedure was vascularized lymph node transfer (31%), followed by preventive lymphatic surgery (27.2%), therapeutic lymphovenous bypass (20.1%), and combined breast and lymphatic reconstruction (9.2%). Overall, most studies were considered level of evidence III (40%) followed by IV (22%), V (17%), II (16%), and I (5%) (Figure 5).

### 3.2. Physiologic Surgery

Of the 37 articles on therapeutic lymphovenous bypass, most articles were categorized into low-level evidence studies (73%), and there were no level I studies (Table 3). The analysis revealed that 36% of studies reported the efficacy of LVB in BCRL patients, 32% on technical variations, 20% on the use of clinical and imaging tools to assist LVB, 4% on robotic-assisted bypass, and others (8%). Interestingly, our analysis revealed that only nine studies reported objective improvements, which ranged from 50 to 100% [32,33,34,35,36,37,38,39,40].

The end-to-end (16) technique was most commonly reported, and studies comparing different types of techniques did not show one technique to be superior to the other [32,41]. Moreover, sleeve-in anastomosis was found to be an effective alternate option for early-stage patients [42]; meanwhile, dermal adipose lymphatic fat wrapping may be useful for late-stage cases with lymphosclerosis refractory to previous LVBs [43]. Two studies emphasized the importance of careful selection of the vessel caliber, number, and appropriate venules to achieve favorable outcomes [44,45]. Additionally, the first human trial on robot-assisted LVB for BCRL treatment in 2022 points to potential future directions for the field [46].

For vascularized lymph node transfer procedures, 57 articles were on VLNT in isolation and 16 on simultaneous VLNT combined with microvascular breast reconstruction (MBR). Of the 14 studies reporting volumetric outcomes, the range of mean volume reduction following isolated VLNT ranged from 22% [47] to 61.8% [48]. Interestingly, in studies at both ends of this range, clinical improvement was noted in 100% of the patients. Similarly, the majority of studies on VLNT were low-level evidence studies (86%): level III evidence (38.6%), IV (26.3), V (21.1%), II (12.3%), and I (1.7%) (Table 3). There was only one level I study, a randomized controlled trial by Dionyssiou et al., which demonstrated a greater mean volume reduction in patients who underwent VLNT (57%) compared to patients undergoing physiotherapy and compression only (18%) [49]. Moreover, in a meta-analysis, Winters et al. revealed a mean circumference reduction of 40.31% between the healthy and affected arms and highlighted the description of compression therapy used pre- and post-operatively across studies as the greatest limitation to assessing volumetric outcomes [50]. The analysis of VLNT donor sites revealed groin (52.3%) nodes as the most used, followed by intra-abdominal (29.2%), supraclavicular (10.8%), submental (4.6%), and thoracic (3.1%). While the inguinal donor site was the most reported in the literature, intra-abdominal-based lymph node transfer, namely the omental or gastroepiploic nodes, has become more prominent since 2017, with 17 articles published on the topic. Five studies reported on donor site morbidity associated with VLNT. Demiri et al. demonstrated a promising 1.6% incidence of donor site lymphedema across 186 patients [51]; meanwhile, others suggest adequate pre-operative lymphatic imaging, knowledge of donor site anatomy, and minimizing donor site trauma as strategies to mitigate such events [51,52,53,54,55].

### 3.3. Preventive Surgery

**Figure 6:**
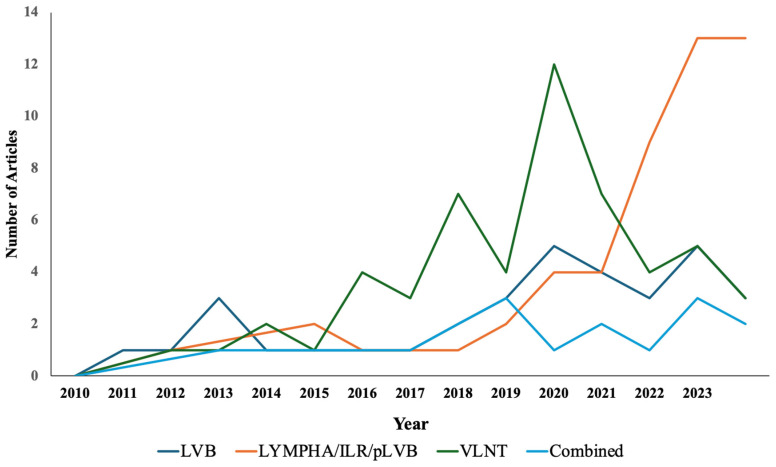
Trend of lymphatic surgery on BCRL patients from 2010 to 2023. LVB = lymphovenous bypass/anastomosis; LYMPHA/ILR/pLVB = lymphatic microsurgical preventive healing approach/immediate lymphatic reconstruction/prophylactic lymphovenous bypass; VLNT = vascularized lymph node transfer.

A total of 50 articles were identified as follows: LYMPHA (32%), ILR (54%), and pLVB (14%). The average number of articles written on this topic dramatically increased from 2.1 per year before 2020 to 11.7 afterward (Figure 6). While the majority of the studies (52%) were level III studies, our search included seven level I studies (14%), including three randomized clinical trials validating the efficacy of preventative lymphatic surgery [23,24,56] (Table 3). There was high heterogeneity in the efficacy of preventive surgery, with reported success in preventing lymphedema ranging from 68.9% [57] to 100% [24], and the size of the study population (11–252) [58,59] was across 32 retrospective and prospective studies. However, Johnson et al. showed a decreased incidence rate of 10.3% compared to 33.4% in patients not having preventive surgery [60]. Of the three preventive surgical techniques, no procedure has emerged as superior to the others when comparing lymphedema prevention success rates between studies; however, no studies from our review specifically investigated the relative superiority of the techniques.

### 3.4. Combined Breast and Lymphatic Reconstruction

Overall, there was a significant increase in the number of articles on this topic in the past 5 years (Figure 6). The search yielded 17 articles on simultaneous lymphatic and breast reconstruction, with 16 exclusively on VLNT and MBR. This procedure category lacked studies from evidence levels I and II, and most studies fall under category IV (47.1%), followed by III (29.4%) and V (23.5%) (Table 3). The use of VLNT in conjunction with MBR was first described by Saaristo et al. in 2012 [61]. Since then, the literature regarding the impact of breast reconstruction itself has been limited, as studies predominantly focus on physiologic techniques. Our search included only two studies on the impact of MBR, with both failing to find a meaningful volume reduction with the addition of MBR; however, one suggested that combining treatments may reduce the need for compression therapy [30,62]. The combination of the superficial iliac artery perforator (SCIP) flap and the deep inferior epigastric artery perforator (DIEP) flap was the most common simultaneous VLNT + MBR procedure (43.8%), followed by the superficial inferior epigastric artery (SIEA) flap and DIEP (18.8%), omental flap and DIEP (18.8%), omental flap-based breast reconstruction (12.4%), and SCIP flap and muscle-sparing transverse rectus abdominis myocutaneous (MS-TRAM) flap (6.4%). There were no studies on combined lymphatic and implant-based breast reconstruction.

## 4. Discussion

As survival has increased with therapeutic advances, patients’ quality of life and survival have become a critical consideration for breast cancer survivors. While there is an increasing demand for preventive and therapeutic lymphatic surgeries in this patient population, there is no consensus or standardized surgical algorithm on how to integrate and combine lymphatic reconstruction as a part of breast reconstruction in patients at risk or with BCRL. Therefore, we aimed to provide a critical appraisal of the current literature by performing a systematic review, and we presented our multi-institutional algorithmic approach to patients at risk or who have BCRL.

Our review highlights important trends in the role of lymphatic surgery in breast cancer patients. While the majority of the literature focused on validating the efficacy of therapeutic physiologic procedures such as LVB and VLNT in patients with BCRL, from 2009 to 2020, there has been a significant shift toward prevention in the past three years. The number of articles on preventive lymphatic surgeries such as LYMPHA/ILR/pLVB has dramatically increased (233%) since 2020. This phenomenon is supported by one randomized clinical trial, which has the highest level of evidence that supports the effectiveness of preventive lymphatic surgery [23]. In addition, recent studies have expanded the aim of their studies to understand the role of BCRL surveillance programs [25,63], bioimpedance as an additional outcome measurement [64], oncologic safety of the procedure [65,66], and variations of the techniques such as the simplified LYMPHA approach [67], the coupler-assisted bypass, and the use of vein grafts [68]. Similarly, our institutions have recognized the importance of surveillance programs, which are a part of our institutional BCRL treatment algorithm.

The shift toward preventive surgery has been complemented by a growing interest in combined lymphatic surgery and breast reconstruction. Previously, simultaneous VLNT and abdominally-based breast reconstruction were popular, given the advantages of a single donor site, decreased operative time, and lack of additional donor sites. However, our study has revealed that there has been a growing interest in using intra-abdominally-based VLNT for either lymphatic or combined lymphatic and breast reconstruction. Three studies evaluated the use of the omental donor site in the settings of VLNT combined with DIEP reconstruction, prophylactic use, and as part of an omental fat-augmented free flap (O-FAFF) [69,70,71]. The major advantages of the omental flap include avoiding iatrogenic lymphedema and bulk, which is useful for patients with axillary contracture or those who desire to use a single donor site for both lymphatic and breast reconstruction. While our study did not identify studies on the combination of lymphatic surgery and prosthetic-based breast reconstruction, this option is a valuable approach for patients who are in early-stage lymphedema, have high anesthesia risk, multiple co-morbidities, and have a history of complicated abdominal surgeries.

Interestingly, our study identified that the popularity of VLNT without simultaneous breast reconstruction has decreased over the years while the number of studies on LVB has been increasing. This phenomenon could be secondary to a change in surgeons’ preferences to perform simultaneous VLNT with autologously-based breast reconstruction, as well as recent technological advances such as high-frequency ultrasound and magnetic resonance lymphangiography (MRL) [72]. While some tools, such as high-frequency ultrasound and ultra-high-frequency ultrasound, are operator-dependent, they have been found to be effective in identifying functional lymphatic vessels and successful at reducing the circumference and volume of the limb [73,74]. The introduction of these tools has expanded the indications of LVB beyond patients with early-stage lymphedema (MDACC stages 1 and 2), and microsurgeons can now offer LVB to those who were previously considered to be poor candidates [75]. This phenomenon illustrates a potential need for instructive courses or training for microsurgeons to be versatile with these imaging tools.

Despite the promising trends identified in our review, the heterogeneity of study designs and outcomes underscores the need for further high-quality research. Our analysis showed that there is a variable range of surgical outcomes (50 to 100% efficacy for LVB; 22 to 61.8% volume reduction for VLNT; 68.9 to 100% efficacy for preventive surgeries). In addition, studies utilized different subjective and objective outcome measures to assess the impact of lymphatic surgeries, preventing high-quality comparative analysis. Furthermore, the majority of the studies included in this review fall under the level III or IV evidence categories, indicating retrospective cohort studies and case series. While these studies provide valuable insights, the lack of randomized controlled trials (level I evidence) limits the ability to make definitive clinical recommendations. Therefore, future studies should aim to include larger, prospective randomized control trials to establish more robust conclusions on the efficacy of lymphatic surgery in BCRL prevention and treatment.

The limitations of our study include the review, data extraction, and designation of evidence level being performed by a single author. Additionally, the heterogeneity of the study designs and lack of high-level evidence studies for certain procedure categories limit the generalizability and comparison of the findings.

## 5. Conclusions

The herein presented study and systematic review of the literature revealed that there is an increasing role of lymphatic surgery in breast cancer patients, with a notable growing interest in the surgical prevention of lymphedema. However, there remains a lack of consensus and standardized algorithms for incorporating lymphatic surgery into breast reconstruction practices. Furthermore, there is a significant lack of high-level evidence studies, and prospective, randomized control trials are needed to validate the effectiveness of different surgical approaches and to establish best practices for integrating lymphatic and breast reconstruction surgery.

Despite the heterogeneity of the studies and controversies, the majority of studies recognize and advocate for prevention, multi-disciplinary care, as well as a combined lymphatic and breast reconstruction approach, and early intervention via surveillance programs. Therefore, an international consensus on treatment protocols and further research is necessary to continue improving patient outcomes in this evolving field.

## Figures and Tables

**Table 1:** American Society of Plastic Surgeons Evidence rating scale for therapeutic studies.

Level of Evidence	Qualifying Studies
I	High-quality, multi-centered or single-centered, randomized control trial with adequate power; or systematic review of these studies
II	Lesser quality, randomized controlled trial; prospective cohort or comparative study; or systematic review of these studies
III	Retrospective cohort or comparative study; case control study; or systematic review of these studies
IV	Case series with pre/post test or only post test
V	Expert opinion developed via consensus process; case report or clinical example; per evidence based on physiology, bench research or “first principles”

**Table 2:** Distribution of studies on breast cancer-related lymphedema by procedure type.

Procedure Category						
	LVB					
Year	Therapeutic	LYMPHA/ILR/pLVB	VLNT	VLNT/LVB + MBR	Review Articles	Articles [n(%)]
1981	1					1 (0.5)
1985	1					1 (0.5)
1986	1					1 (0.5)
1988	1					1 (0.5)
2009			1			1 (0.5)
2010	1					1 (0.5)
2011	1	1	1			3 (1.6)
2012	3		1	1		5 (2.7)
2013	1		2		1	4 (2.2)
2014		2	1			3 (1.6)
2015	1	1	4	1	2	9 (4.9)
2016	1		3	1	1	6 (3.3)
2017	2	1	7	2	1	13 (7.1)
2018	3	2	4	3	2	14 (7.6)
2019	5	4	12	1	1	23 (12.5)
2020	4	4	7	2	2	19 (10.3)
2021	3	9	5	1	3	21 (11.4)
2022	5	13	6	3	4	31 (16.8)
2023	3	13	3	2	6	27 (14.7)
Total [n(%)]	37 (20.1)	50 (27.2)	57 (31)	17 (9.2)	23 (12.5)	184

LVB = lymphovenous bypass/anastomosis; LYMPHA/ILR/pLVB = lymphatic microsurgical preventive healing approach/immediate lymphatic reconstruction/prophylactic lymphovenous bypass; VLNT = vascularized lymph node transfer; MBR = microvascular breast reconstruction.

**Table 3:** Analysis of study procedure type based on the level of evidence.

	Number of Articles
**LVB**	
I	
II	10
III	12
IV	8
V	7
**LYMPHA/pLVB/ILR**	
I	7
II	8
III	26
IV	4
V	5
**VLNT**	
I	1
II	7
III	22
IV	15
V	12
**Combined**	
I	
II	
III	5
IV	8
V	4

LVB = lymphovenous bypass/anastomosis; LYMPHA/ILR/pLVB = lymphatic microsurgical preventive healing approach/immediate lymphatic reconstruction/prophylactic lymphovenous bypass; VLNT = vascularized lymph node transfer.
